# Bibliometric analysis of cerebral organoids and diseases in the last 10 years

**DOI:** 10.1002/ibra.12139

**Published:** 2023-11-30

**Authors:** Bo‐Yan Luo, Ke‐Qian Liu, Ji‐Sheng Fan

**Affiliations:** ^1^ Interdisciplinary Research Center on Biology and Chemistry, Shanghai Institute of Organic Chemistry Chinese Academy of Science Shanghai China; ^2^ Science Computer and Engineering of University of South Australia Adelaide South Australia Australia

**Keywords:** bibliometric analysis, cerebral organoids, induced pluripotent stem cells, neurological diseases

## Abstract

Cerebral organoids have emerged as a powerful tool for mirroring the brain developmental processes and replicating its unique physiology. This bibliometric analysis aims to delineate the burgeoning trends in the application of cerebral organoids in disease research and offer insights for future investigations. We screened all relevant literature from the Web of Science on cerebral organoids in disease research during the period 2013–2022 and analyzed the research trends in the field using VOSviewer, CiteSpace, and Scimago Graphica software. According to the search strategy, 592 articles were screened out. The United States of America (USA) was the most productive, followed by China and Germany. The top nine institutions in terms of the number of publications include Canada and the United States, with the University of California, San Diego (USA), having the highest number of publications. The International Journal of Molecular Sciences was the most productive journal. Knoblich, Juergen A., and Lancaster, Madeline A. published the highest number of articles. Keyword cluster analysis showed that current research trends focused more on induced pluripotent stem cells to construct organoid models of cerebral diseases and the exploration of their mechanisms and therapeutic modalities. This study provides a comprehensive summary and analysis of global research trends in the field of cerebral organoids in diseases. In the past decade, the number of high‐quality papers in this field has increased significantly, and cerebral organoids provide hope for simulating nervous system diseases (such as Alzheimer's disease).

## Graphical abstract

This review explores the progress of research on cerebral organoids in the last 10 years, mainly using bibliometric methods. The Web of Science (WOS) database was searched for literature related to cerebral organoids and diseases published from 2013 to 2023 using the keywords “cerebral organoids” and “diseases.” Research trends in the field were analyzed using VOSviewer, CiteSpace, and Scimago Graphica software. The methodology was explored to derive the current research hotspots in the field of cerebral organoids and future directions. The current research trend focuses on the use of stem cells to construct brain organoids, and then delves into the mechanisms and treatments of neurological diseases. In addition to this, cerebral organoids are also used for the screening of drugs for neurological diseases, and this application will accelerate the development of drugs for neurological diseases. This article analyzes the last 10 years of research on cerebral organoids and diseases, which will help researchers understand the current status of the field, anchor research directions, and provide a reference base for advancing the field.

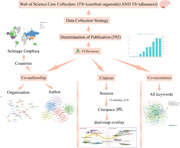

## 1 INTRODUCTION

The brain, being the largest and most intricate organ, shows intricate neural activity, primarily responsible for motor, sensory, auditory, and visual functions as well as higher cognitive functions like consciousness and memory.^1^, ^2^ In the past, due to ethical and moral restrictions and the origin of human brain tissue, human research on the brain was usually limited to the common characteristics of mammals and other vertebrates. The search for the unique structure and function of the developing human brain remains at a standstill. However, the advent of organoids has enabled researchers to overcome this limitation. Organoids are three‐dimensional (3D) cell culture constructs that replicate organ‐specific structures. They are created by inoculating stem cells in stromal gel or basement membrane extracts in the presence of a specific mixture of cytokines.^3^ Organoid models have a more stable genetic and phenotypic profile and a richer range of cell types in vitro than traditional cellular models.^4^, ^5^ In addition, organoids can possibly reproduce some organ functions in vitro. Due to the development of stem cell technology, researchers use induced pluripotent stem cells (iPSC) to induce differentiation into brain tissue and even brain organs from a 3D perspective.^6^, ^7^ In the past few years, there has been considerable progress in cerebral organoid technology from providing visualization of a featureless whole‐brain organ to a regionally characterized brain organ such as the cortex, midbrain, hippocampus, cerebellum, and spinal cord, and to the vascularization of brain organs.^8^, ^9^, ^10^, ^11^, ^12^, ^13^ Currently, cerebral organoids, as a good model for in vitro study of human brain‐related diseases, are widely used in the study of neurodegenerative diseases (NDDs), such as Alzheimer's disease (AD),^14^ Parkinson's disease (PD),^15^ microcephaly,^16^ autism spectrum disorders (ASD),^17^ and other brain diseases. By simulating the intricate process of human neurodevelopment and the progression of neurodegenerative diseases, we can uncover crucial disease targets. This approach enables us to develop more precise and effective treatments.

As a method to study the characteristics and trends of the literature, bibliometrics analysis focuses on evaluating academic productivity in different fields and summarizes academic frontiers and hotspots based on scientific literature databases and metrological characteristics. Compared to traditional reviews and meta‐analyses, bibliometric analysis provides a deeper level of analysis that cannot be achieved using other methods.^18^ In addition, bibliometric analysis allows for the estimation of the contributions and collaborations of different countries, institutions, journals, and authors using qualitative and quantitative methods, as well as analysis of the status and direction of research in different fields through the co‐occurrence of keywords.^19^ Currently, bibliometric analysis is widely used in medical fields, including exploration of cross‐sectional and longitudinal scientific productivity in intestinal‐like organs,^20^ scientific outputs, and emerging themes related to esketamine,^21^ and stem cell extracellular vesicles for diabetes.^22^

With the great progress made in the study of cerebral organoids in diseases, the number of related publications has increased dramatically in recent years. Although several reviews have systematically summarized the research on cerebral organoid‐related diseases from different aspects,^23^, ^24^, ^25^ there is currently no comprehensive bibliometric analysis available in the literature that examines the current status and trends of research in this field. In this study, the literature on cerebral organoids and diseases on the Web of Science (WOS) database from 2013 to 2022 is analyzed and a statistical analysis on article output and institutions is carried out, with the aim of aiding researchers in identifying global research hotspots and directions in this field to further advance research progress.

## 2 MATERIALS AND METHODS

### 2.1 Data collection

**Figure 1:**
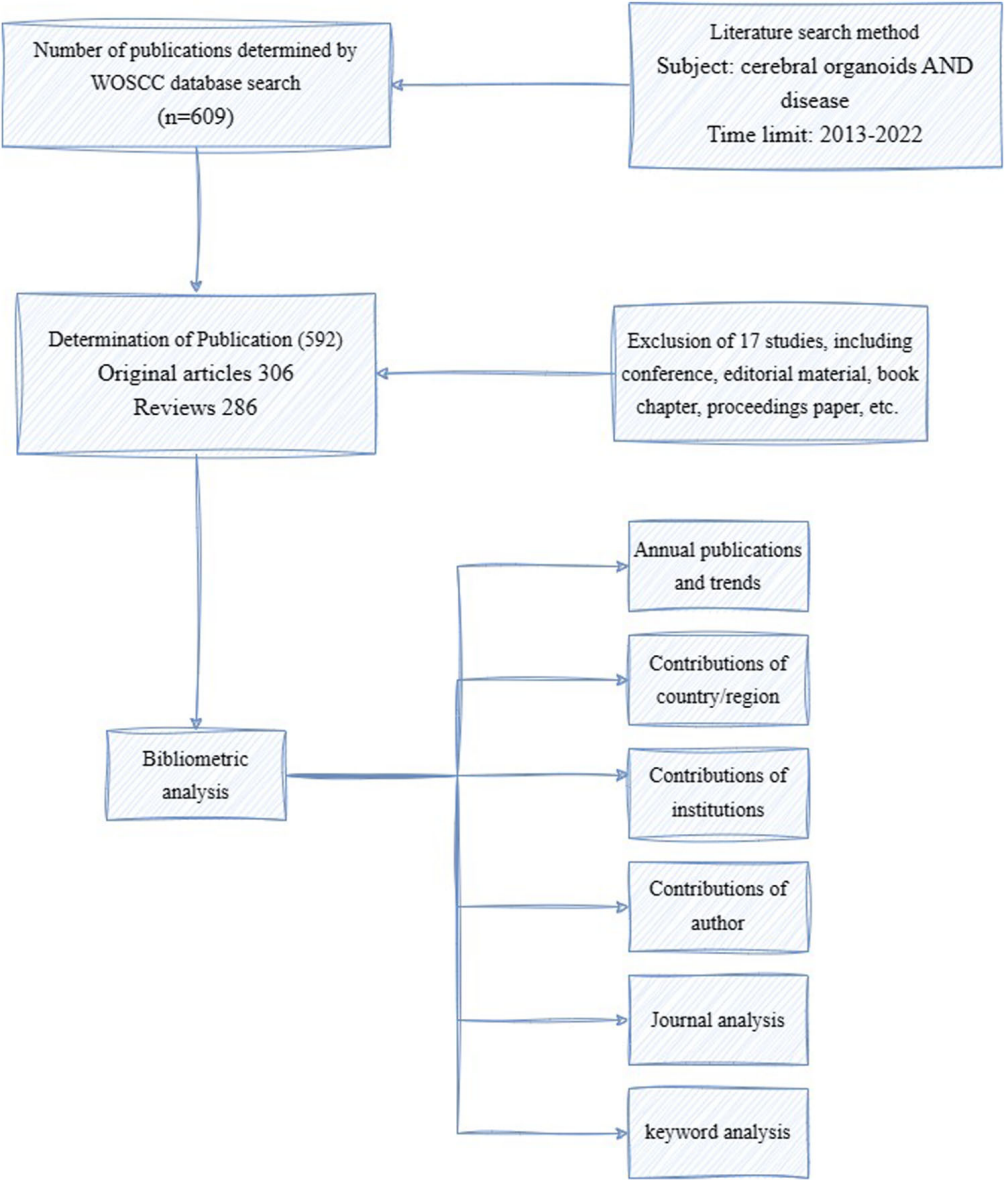
Flow diagram of literature identification. *n* represents the number of articles; WOSCC, Web of Science Core Collection. [Color figure can be viewed at wileyonlinelibrary.com]

WOS has been accepted by many researchers as a high‐quality digital literature resource database and was considered to be the most suitable database for bibliometrics analysis.^26^ Therefore, the WOS Core Collection (WOSCC) was chosen as the data source in this study. The index was chosen as Science Citation Index Expanded to ensure comprehensiveness and accuracy of the search data. The search strategy was as follows (Figure 1): TS = (cerebral organoids) AND TS = (diseases) AND Publication Date = (January 1, 2013 to December 31, 2022). The type of literature selected included original articles and reviews. A total of 609 articles were searched. Then, the papers were screened (Figure 1) and 592 valid papers (comprising 306 original articles and 286 reviews) were identified. The full data (including countries, institutions, authors, keywords, source journals, and citations) were searched on the WOSCC using the strategy described above. The literature search was conducted on March 4, 2023 to prevent possible bias caused by frequent updates to the database.

### 2.2 Data analysis and visualization

Based on the data extracted from WOS, we first analyzed publication and citation trends for cerebral organoids and diseases and visualized them using Microsoft Excel 2019 (Microsoft Corporation). We then used VOSviewer 1.6.18 (Leiden University), CiteSpace 6.1.R2 (Chaomei Chen, Drexel University), and SCImago Graphica 1.0.24 (SCImago Lab; SRG S.L. Company) for bibliometric analysis, including collaborative network analysis of national and institutional literature, cocitation analysis of literature, and keyword co‐occurrence analysis. The latest version of the Journal Citation Reports (JCR) was used to obtain the latest impact factors (IF). The H‐index was obtained using the Scimago Journal & Country Rank (https://www.scimagojr.com/).

## 3 RESULTS

### 3.1 Annual publications and trends

**Figure 2:**
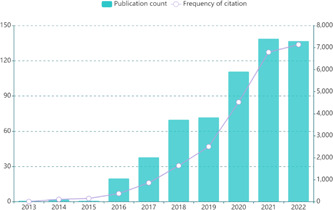
Global trends and frequency of citation related to cerebral organoids and disease research from 2013 to 2022. [Color figure can be viewed at wileyonlinelibrary.com]

According to the search strategy, 592 papers related to cerebral organoids and diseases were retrieved and included in the final bibliometrics analysis. On the whole, the general trend of published research in this field is a significant increase year by year (Figure 2); especially after 2017, the number of publications has increased rapidly. The published research in the field of cerebral organoids and diseases stabilized at over 120 in both 2021 and 2022, indicating that this field has been receiving increasing scholarly attention in recent years and has become a key focus in the field of medical research. Notably, citations to articles in this area showed a more significant increase from 2019 to 2021 (2504–6791 citations), indicating the widespread interest in the use of brain‐based organs for disease research in general.

### 3.2 Contribution of countries or regions

**Figure 3:**
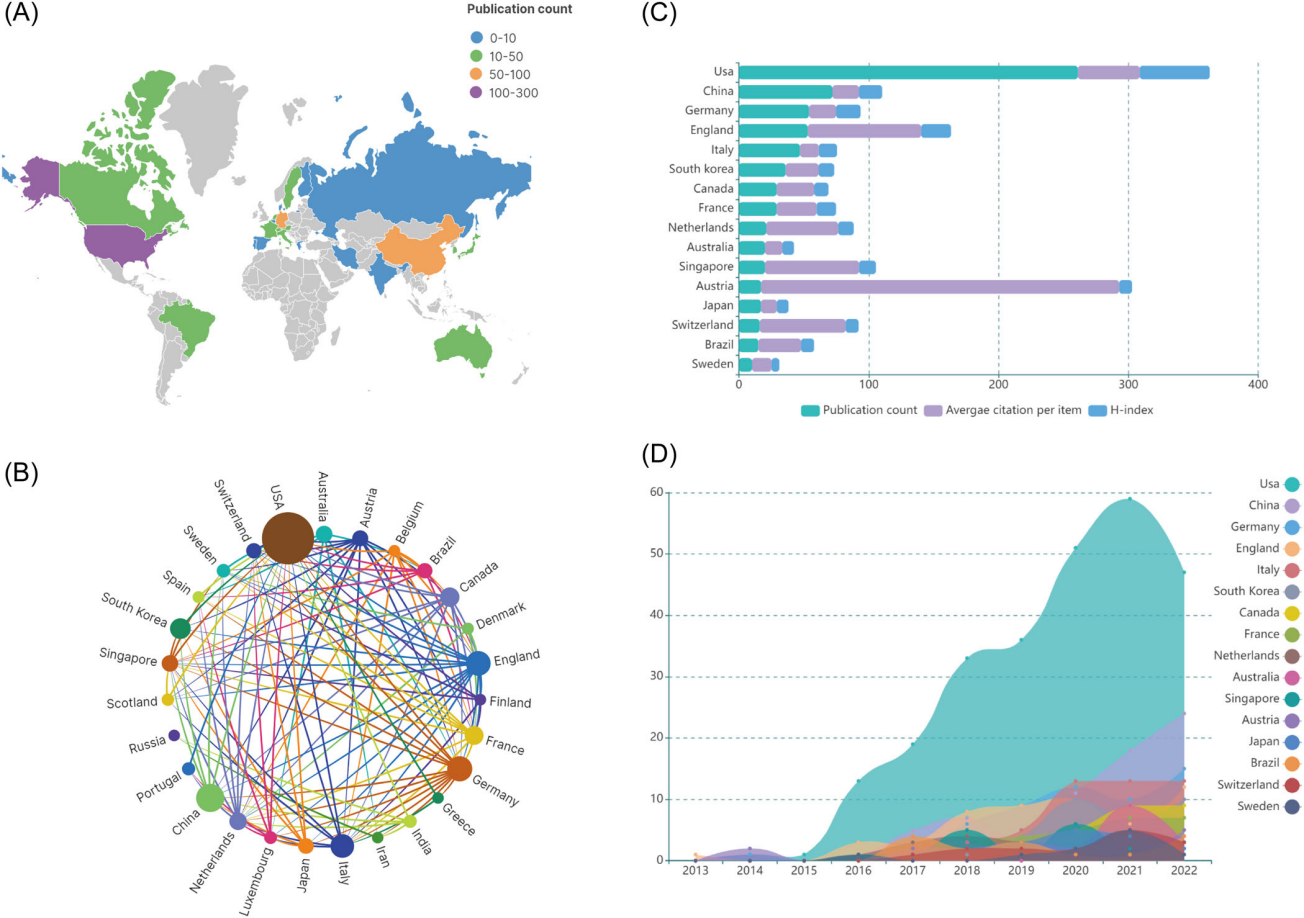
Contribution of countries/regions to the field of cerebral organoids and disease research. (A) World map showing the global distribution of cerebral organoids and disease research. Different countries are indicated by different colors based on the number of articles published. (B) Distribution and international cooperation of countries/regions involved in cerebral organoids and disease research. The thickness of the line reflects the frequency of the cooperation; the thicker the line, the stronger the cooperation. (C) Total number of publication counts, average citation per item, and H‐index of countries that contributed more than 10 publications in this field. (D) Growth trends in the publication count of the countries/regions that contributed more than 10 publications in the field of cerebral organoids and disease research from 2013 to 2022. [Color figure can be viewed at wileyonlinelibrary.com]

To further understand which country has made the greatest contribution to the field of cerebral organoids and disease research, in this study, the volume of publications from 49 countries was analyzed. On using VOSviewer, it was found that 27 of these countries carried out at least five studies. The United States of America (USA) had the largest number of publications on cerebral organoids and diseases, with 260 articles, followed by China (70), Germany (54), England (52), and others (Figure 3A). The larger the node of the circle in Figure 3B, the more researchers from that country collaborate with researchers from other countries, and researchers from the United States collaborate the most with researchers from other countries, far more than any other country. In terms of the average frequency of citations, the top three countries were Austria (275.82 citations), England (87.34 citations), and Singapore (72.50 citations) (Figure 3C). In addition, on the basis of the time of publication, as shown in Figure 3D, it was found that England published the first article in the field in 2013, followed by the United States, Germany, and Austria in 2014. Asian countries, including China, started to publish their research results after 2016. Furthermore, in terms of the year of concentration of country‐specific publications, the United States showed an explosive growth after 2015, while most of the articles from China, Italy, and Germany were published after 2020. Thus, the contribution of the United States to the field cannot be ignored.

### 3.3 Contribution of institutions

**Figure 4:**
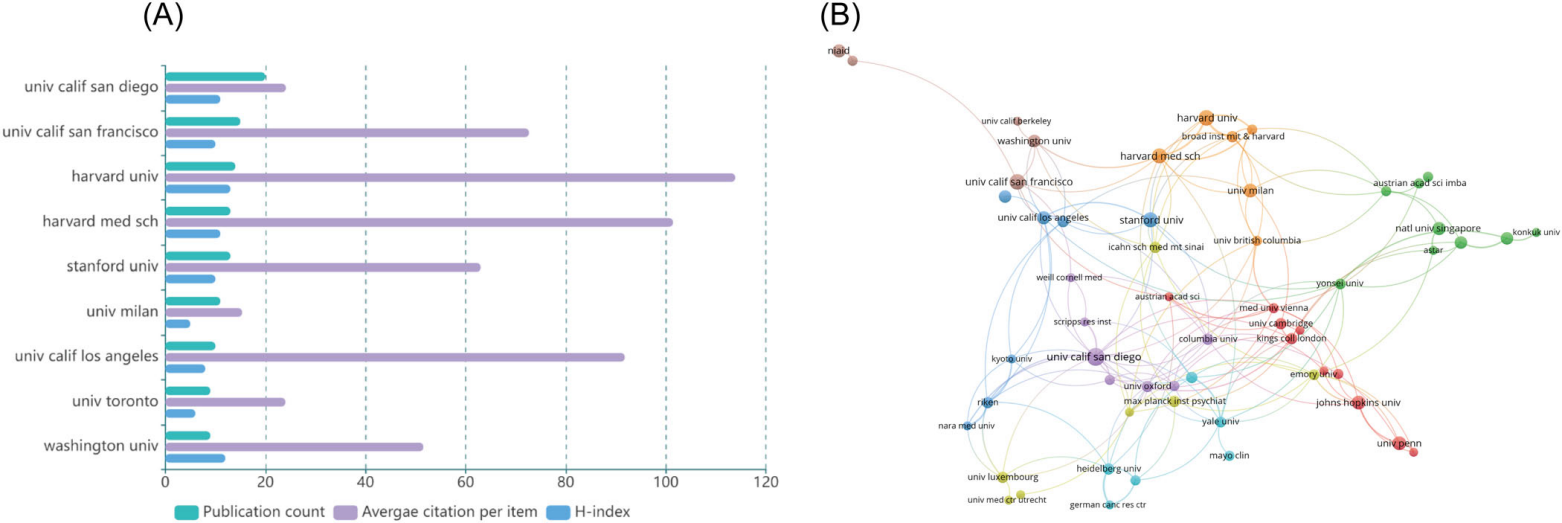
Contribution of institutions in the field of cerebral organoids and disease research. (A) Total number of publications counts, average citation per item, and H‐index of institutions that contributed a minimum of nine papers in this field. (B) Mapping of the coauthorship analysis among the top 55 most productive institutions in cerebral organoids and disease research according to VOSviewer. Each node represents an institution, and the node size indicates the number of publications. The connection between the nodes represents a coauthorship relationship, and the thickness of the lines indicates strength. [Color figure can be viewed at wileyonlinelibrary.com]

A total of 887 different institutions published research on cerebral organoids and diseases. Nine of these institutions published at least nine articles, all in the United States, except for the University of Toronto (UofT), in Canada. The University of California, San Diego (UCSD), had the highest number of articles, with 19, followed by the University of California, San Francisco (UCSF), with 15 (Figure 4A). In terms of average citations, Harvard University was cited 113.9 times, much more than any other institution (Figure 4A).

In addition, using VOSviewer, complex collaborative relationships between different institutions were analyzed. First, we set a threshold of six for the minimum number of institutional documents and then presented the top 55 institutions that met the threshold in the coauthorship analysis network (Figure 4B). We found 306 links to the top 55 productive institutions, with the UCSD in the United States having the strongest links, suggesting that this institution has a strong presence in the field of cerebral organoid use in brain disease research (Figure 4B).

### 3.4 Contribution of authors

**Figure 5:**
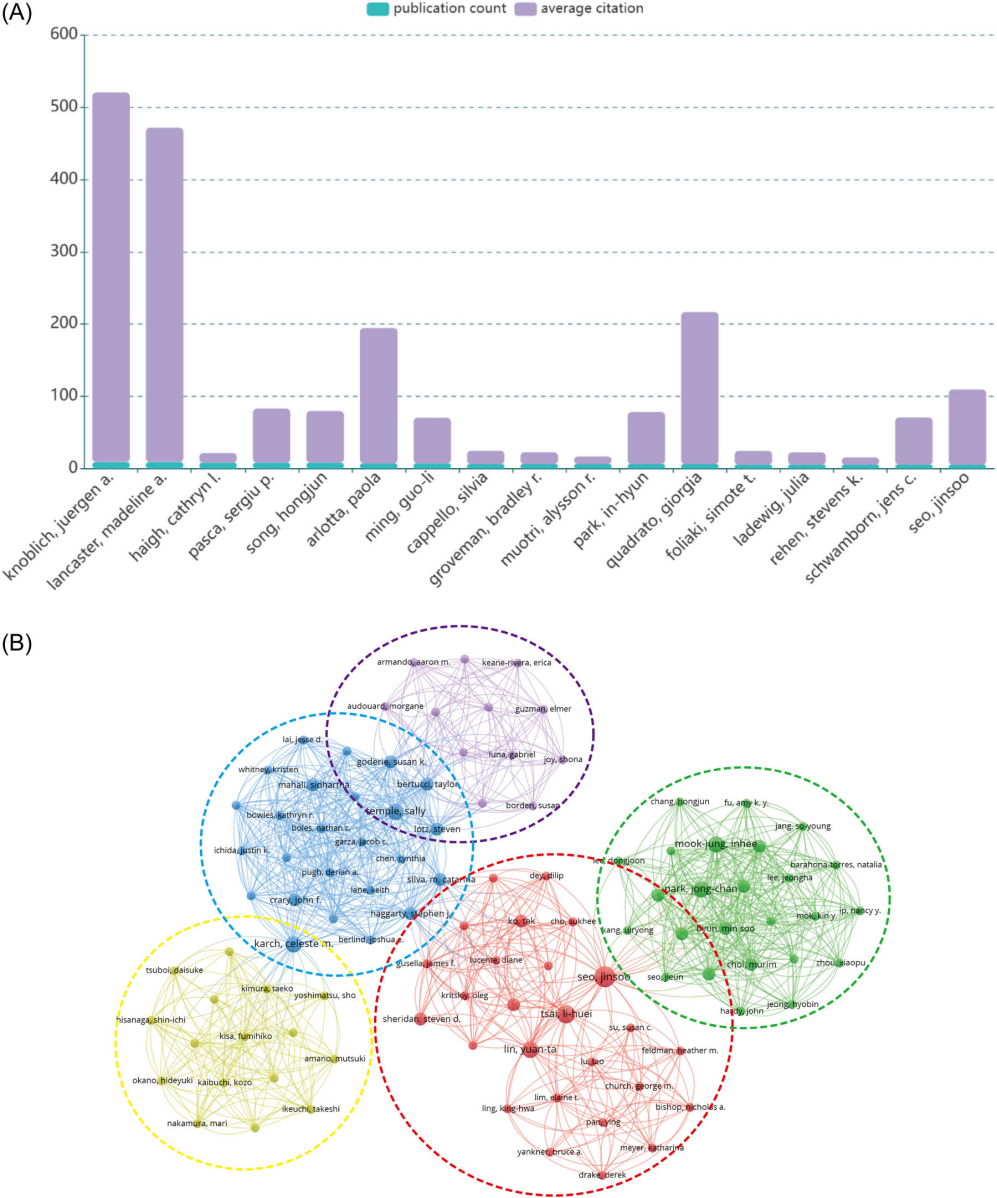
Contribution of authors in the field of cerebral organoid and disease research. (A) Total number of publication counts and average citation per item of authors who contributed at least five articles in this field. (B) Mapping of the coauthorship analysis among the authors who published at least one paper on cerebral organoids and disease research according to VOSviewer. Each node represents an institution, and the node size indicates the number of publications. The connection between the nodes represents a coauthorship relationship, and the thickness of the lines indicates strength (TLS). [Color figure can be viewed at wileyonlinelibrary.com]

A total of 3143 authors were part of studies on cerebral organoids and diseases, of whom only 17 published at least five papers (Figure 5A). Knoblich, Juergen A. and Lancaster, Madeline A. from the Institute of Molecular Biotechnology (IMBA) were the most productive and influential authors in the field of cerebral organoids and diseases, both publishing nine papers (Figure 5A), with H‐indexes of 68 and 33, respectively. The average number of citations to an article was also an important indicator of an author's influence. The first and second most frequently cited authors were still Knoblich, Juergen A. and Lancaster, Madeline A., with average citations of 511.56 and 462.78, respectively, about twice the average citations of the rest of the authors. This indicated that these two scholars from the same institution have achieved great success and recognition in the field of cerebral organoid use in brain disease research.

Subsequently, a coauthorship network analysis was performed using VOSviewer. The threshold for the minimum number publication of author was set at one, resulting in the filtration of a total of 104 interconnected authors, yielding five distinct clusters (Figure 5B). In Figure 5B, the importance of a node in the network was quantitatively determined by its total link strength (TLS) with other nodes. The results of the coauthorship analysis showed that Karch, Celeste m (TLS = 42), Seo, Jinsoo (TLS = 47), Temple, Sally (TLS = 41), Mahali, Sidhartha (TLS = 26), and Mook‐jung, Inhee (TLS = 34) were at the center of the cluster (Figure 5B). The active strength of collaboration between authors in the field of cerebral organoids in disease research was mostly consistent, with the strength of connection between authors in the same cluster or not ranging from 1 to 3, with the strongest connection between Mook‐jung, Inhee and Park, Jong‐chan (strength of connection: 3).

### 3.5 Journal analysis

**Figure 6:**
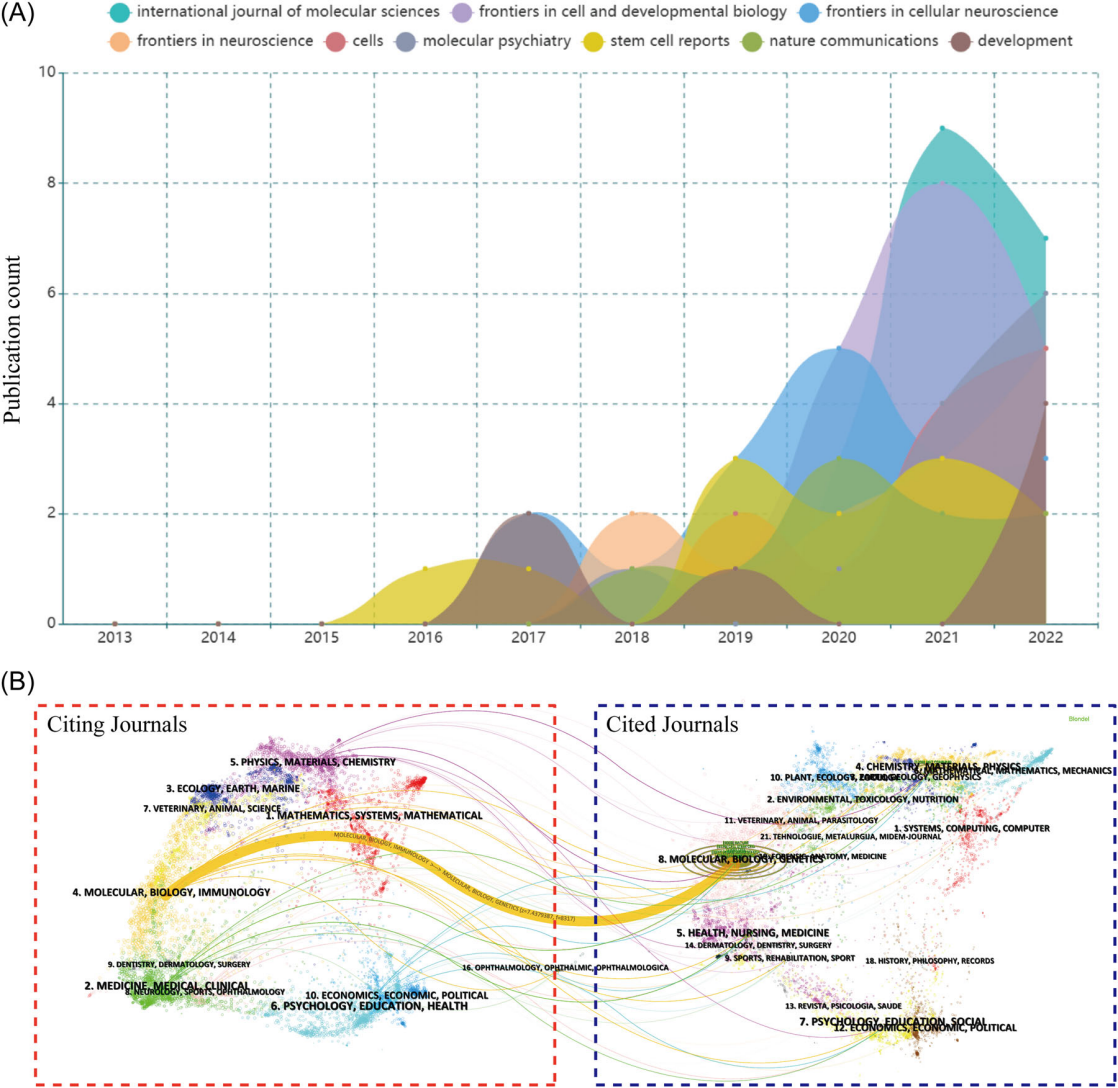
Contribution of journals in the field of cerebral organoids and disease research. (A) Growth trends in the number of publications of the top nine productive journals in cerebral organoids and disease research from 2013 to 2022. (B) Dual‐map overlay of the journals on cerebral organoids and disease research generated by CiteSpace. The labels represent different research subjects covered by the journals. The citation journals are on the left side, while the other side of the map shows the cited journals. Different‐colored lines correspond to the different paths of references, beginning with the citation map and ending at the cited map. [Color figure can be viewed at wileyonlinelibrary.com]

Journal analysis helps to identify the top journals in the field of cerebral organoid use in brain diseases. A total of 284 journals published relevant research in this field. The top nine journals published 121 papers on cerebral organoids and diseases, accounting for 20.44% of the total of 592 publications (Table 1). The International Journal of Molecular Sciences (IF = 5.6, 2022) was the most productive journal, publishing 20 papers on cerebral organoids and diseases. According to the JCR 2022 standards, most of the top nine active journals were classified as Q1 or Q2. Interestingly, there were no published papers in this field in the top nine journals during the 2013–2015 period. However, these journals have published more than half of the research in this field in the past 5 years. This suggests that the popularity of this field increased in recent years, before the major journals began to actively take interest in this field of research (Figure 6A). Figure 6B shows a dual‐map overlay of the journals on cerebral organoids used in brain disease research, with citation frequencies on the left and cited frequencies on the right. Overall, the published studies mainly targeted journals in one field: molecular, biology, and immunology, whereas the most cited publications originated from journals covering molecular, biology, and genetics. We can observe a high degree of overlap between the themes of the citation and cited journals, which is indicative of the future direction of this research.

**Table 1:** The top nine journals for cerebral organoids and disease research.

Rank	Journal	Publication count	IF (2022)	JCR 2022	Total citations	Average citation per item
1	International Journal of Molecular Sciences	20	5.6	Q1	199	9.95
2	Frontiers in Cell and Developmental Biology	19	5.5	Q1	245	12.89
3	Frontiers in Cellular Neuroscience	17	5.3	Q1	221	13.00
4	Frontiers in Neuroscience	13	4.3	Q2	138	10.62
5	Cells	12	6.0	Q2	80	6.67
6	Molecular Psychiatry	12	11.0	Q1	366	30.50
7	Stem Cell Reports	12	5.9	Q2	570	47.50
8	Nature Communications	9	16.6	Q1	602	66.89
9	Development	7	4.6	Q1	571	81.57

Abbreviations: IF, impact factor; JCR, journal citation reports.

### 3.6 Keyword analysis

Keywords represent the central themes of a publication, and co‐occurrence analysis of keywords helps us to gain an overall understanding of hot topics and current advances and intrinsic connections. Using VOSviewer, with a threshold set at a minimum of 10 occurrences of the keywords in the titles and abstracts of the included literature, a total of 121 keywords were selected, and the top 20 most frequent keywords are presented in Table 2. This suggested that brain organoid research was strongly related to stem cells, which is in line with existing research directions (Table 2).

**Table 2:** Top 20 keywords in terms of frequency of occurrence.

Rank	Keyword	Occurrence	TLS
1	Cerebral organoids	404	2287
2	Pluripotent stem cells	230	1494
3	Differentiation	117	768
4	Self‐organization	108	779
5	Organoids	89	543
6	In vitro	86	558
7	Model	85	467
8	Generation	84	540
9	Neurons	84	543
10	Brain organoids	68	487
11	Neural progenitors	63	424
12	Disease	61	328
13	Disease modeling	54	426
14	Directed differentiation	53	370
15	Human brain development	48	309
16	Brain	46	227
17	Expression	43	225
18	Culture	42	303
19	Human es	41	335
20	Stem cells	38	277

Abbreviation: TLS, total link strength.

**Figure 7:**
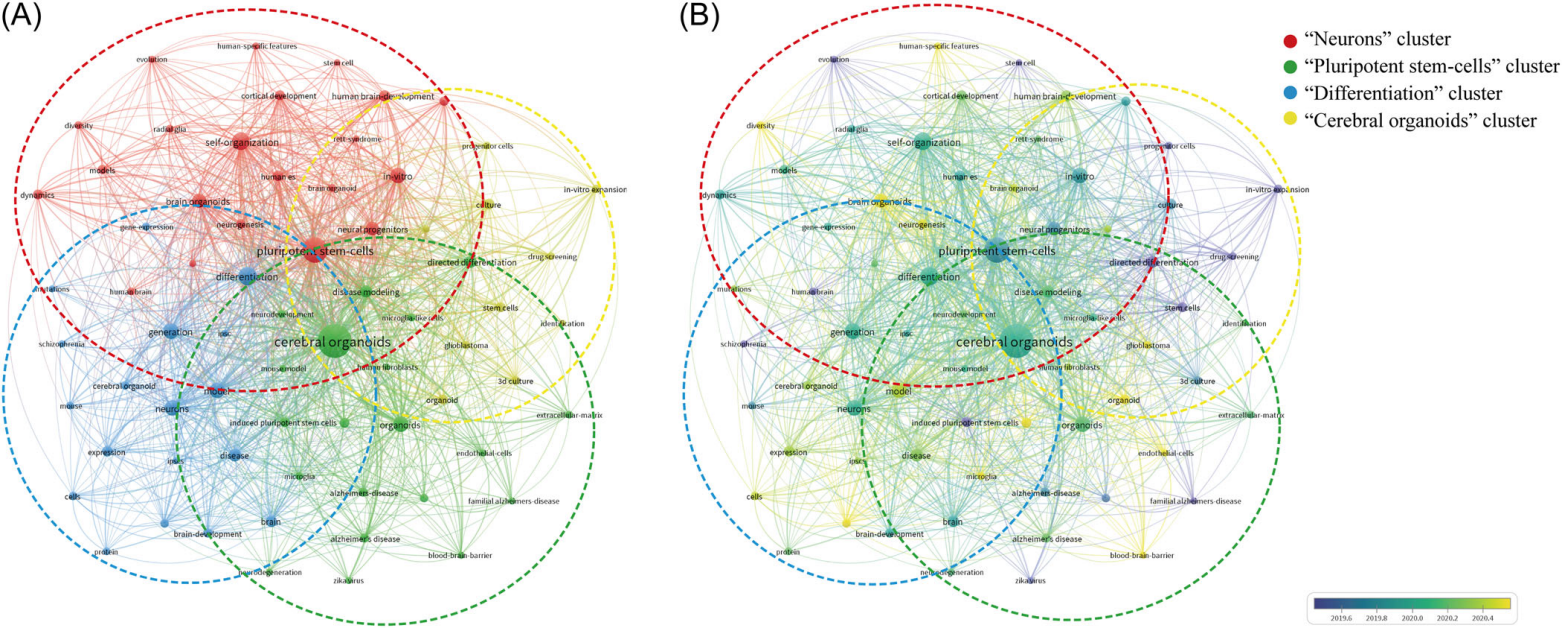
Contribution of keywords in the field of cerebral organoids and disease research. (A) Keyword co‐occurrence analysis on cerebral organoids and disease research using VOS viewer. (B) Overlay visualization of the keyword co‐occurrence analysis. The purple nodes represent the keywords appearing earlier, whereas the yellow nodes show the recently appearing keywords. [Color figure can be viewed at wileyonlinelibrary.com]

In the co‐occurrence network diagram (Figure 7A), we divided the identified keywords into four main different clusters: the first cluster focused on neurons related to disease modeling, directed differentiation (red), the second cluster focused on pluripotent stem cells related to self‐organization, in vitro (green), the third cluster focused on differentiation related to modeling, generation (blue), and the fourth cluster focused on cerebral organoids (yellow) (Figure 7B). The time zone view of the keywords, shown in Figure 7B, shows the evolution of the high‐frequency keywords, as is clear from the fact that the keyword “cerebral organoids” appears on average in 2020, while “disease” basically appears in the same year. This indicated that the application of cerebral organoids in disease has indeed been a trend in recent years.

## 4 DISCUSSION AND PERSPECTIVES

### 4.1 Bibliometric results on cerebral organoids and diseases

In this study, the bibliometrics analysis method was used to analyze the research results of cerebral organoids and diseases from 2013 to 2022. The analysis found a rapid increase in the total number of publications in this field as well as in the frequency of citations, especially in the last 5 years, indicating that the topic of cerebral organoid use in brain diseases has become a trend for current research. Cerebral organoids are currently used to study disease processes in two main ways: to determine the influence of known risk factors on disease and to identify the mechanisms of the disease caused by known causes.^27^ With the increase in genome‐wide association studies and the widespread use of diagnostic genome sequencing, a large number of genetic variants associated with diseases have been identified, such as ASD in neurodevelopmental disorders,^28^ and many single‐gene genetic disorders like neurodegenerative diseases.^29^ However, the causal relationship among these risk factors, disease occurrence, and the course of disease progression are still poorly understood. Hence, cerebral organoids present an optimal solution to study the causal relationship between these risk variants and defined cellular phenotypes, along with identification of the mechanisms underlying pathogenic mutations in human tissues.^30^

The keyword co‐occurrence analysis revealed that pluripotent stem‐cells (PSCs) and cerebral organoids are the largest clusters, demonstrating that these keywords emerged in 2020 on average, and the emergence of PSCs precedes that of cerebral organoids. Human embryonic stem cells first appeared in 1998 when they were isolated from human blastocysts by scientists; this laid the foundation for the emergence and development of organoid technology.^31^ Based on this literature, PSCs can differentiate into motor neurons, astrocytes, and oligodendrocytes in response to various inducers, and then these heterogeneous neural cell populations obtained through differentiation can successfully build cerebral organoids,^7^, ^32^, ^33^ which can accurately mimic the in vivo environment, cell–cell, and cell–extracellular matrix interactions;^34^, ^35^ also, they represent a good model for accurate investigations into the mechanisms of brain diseases.

According to publication outputs, we identified the top nine influential journals in cerebral organoids and diseases, all of which have an IF of four or higher, which means that the influence of this field has spread globally. Since 2018, relevant journals in the field have been publishing disease modeling derived from human iPSCs and have included articles on modeling of related psychiatric disorders,^36^ such as NDDs, AD, PD,^37^ and ASD,^38^ neurological disorders due to Zika virus infection,^39^ and neurodevelopmental disorders due to genetic mutations.^40^ These data indicate that brain organoid disease models are already able to reproduce the development and disease of the most complex of human tissues, and are increasingly being applied for all kinds of brain diseases. Thus, it will be possible to continue to promote the use of brain organoids to explore brain diseases for a long time to come.^23^

Subsequently, by analyzing the distribution of authors of publications in terms of their affiliations and countries, we gained a deeper understanding of the field of cerebral organoids and diseases. First, we can see that Austria has the highest citation frequency (276.35), while the two authors Knoblich, Juergen A. and Lancaster, Madeline A. from Austria were the most productive and had the highest number of publications. Further exploration of their publications revealed that the two researchers and their team had already proposed in 2013 that the use of cerebral organoids could recapitulate the development and mechanisms of diseases of the human brain, established a protocol for generating 3D brain tissue the following year, and proposed in 2017 that fused cerebral organoids could be used to analyze complex neurodevelopmental defects and test the possibility of potential therapeutic compounds.^7^, ^27^, ^41^ This has an indelible impact at every critical developmental point in cerebral organoids mimicking neurological disorders.

In addition, the authors at the center of the collaborative cluster in the coauthorship analysis are from American and European institutions, suggesting that the field is still dominated by researchers from the United States and Europe, with Seo, Jinsoo from the Massachusetts Institute of Technology (MIT) in the United States being the most prominent. Seo, Jinsoo has been working on the development of an AD pathology model system with high predictive validity since 2016, not only finding the consistent incidence of AD pathology among multiple familial AD strains carrying different mutations but also finding that treatment with β‐ and γ‐secretase inhibitors significantly reduced amyloid and tau.^42^ In‐depth studies revealed an important role of p25/Cdk5 in tau pathology,^43^ culminating in the successful development of an efficient, web‐based drug screening platform for AD.^44^ This will greatly aid researchers in screening therapeutic drugs in the field of AD and speed up the feasibility of effective treatments. This is only the result of cooperation between some countries. There is no doubt that with stronger cooperation between institutions across many countries, progress of brain organic matter use in brain disease research will be faster.

### 4.2 Perspectives of cerebral organoids in neurological diseases

A bibliometric analysis shows that the use of these models and their continuous improvement have exploded in this field since the neural‐like organs were first put forward, because we have a very large research group to promote the in‐depth development of this field. In 2013, Lancaster et al. reported for the first time an ongoing human pluripotent stem cell‐derived 3D organoid culture system called a brain organoid.^7^ Then, a researcher succeeded in achieving direct differentiation into individual brain regions by manipulating growth factor signals in stem cell differentiation,^45^, ^46^ confirming the feasibility of the model. With the optimization of 3D culture systems year by year, this has facilitated the study of cerebral organoids resembling different regions of the human brain, enabling the study of specific brain regions such as the midbrain, hypothalamus, and cerebellar‐like organs.^8^, ^9^, ^47^ This was followed by attempts to combine these separate local cerebral organoids to explore the mechanisms and complex processes of brain development and neurological disorders.^27^, ^48^, ^49^ While molecular analysts have used single‐cell RNA sequencing to demonstrate the remarkable similarity between in vitro and in vivo developmental trajectories of cerebral organoids,^50^, ^51^, ^52^, ^53^ electrophysiology researchers have also uncovered the great potential of cerebral organoids to form relatively mature neuronal networks.^54^, ^55^, ^56^ Also, Cakir et al. successfully constructed complex vascular‐like networks in cerebral organoids,^12^ and vascularized cerebral organoids more accurately reflect the physiological state of the human brain, which provides a more reliable model for studying higher brain function in vitro. Thus, many disease‐associated genes or molecules that have been studied in cellular or animal experiments can now be studied in depth using cerebral organoids, which can be used not only to explore the pathogenesis and development of various brain diseases but also to provide a new avenue for the development of novel therapeutic agents for diseases,^37^, ^57^, ^58^ which is a promising direction for research.

### 4.3 Cerebral organoids in ASD research

To date, organoid models have been successfully applied to study events of neural precursor cell dysfunction occurring in the early stages of brain development, including precursor cell abnormalities of Zika virus infection and its resulting microcephaly‐related phenotypes,^59^, ^60^ which were the first developmental disorders to be studied in organoids.^7^ The use of organoids successfully revealed that the Zika virus disrupts the apical junctions of radial glial cells (RGCs) through the protein NS2A,^61^ causing RGCs to proliferate less, produce fewer neurons, and produce apoptosis,^9^, ^62^ leading to disruption of ventricular zone structures and RGC scaffolds and tissue structural disruption,^63^ ultimately producing a microcephalic phenotype.^64^, ^65^ These studies demonstrate that organoids can be used to replicate the complex tissue phenotypes that occur as a result of viral infection and provide mechanistic insights into the effects of infection. Organoids produced by patients with severe idiopathic ASD have also been used to explore the causes of progenitor overproliferation and excessive production of GABAergic neurons in this complex disorder. However, due to the heterogeneity of the etiology of autism spectrum disorders, it is difficult to create organoid models that accurately capture the characteristics of the disorder in real time and understanding of the disease, and a trade‐off between the clinical presentation and the associated molecular basis is needed to generate representative brain organoid models of ASD,^66^ which is where our future efforts should be directed.

### 4.4 Cerebral organoids in AD research

In addition, AD, the most common neurodegenerative disease present worldwide, has been well studied in organoid models. The earliest models of human‐derived AD pathogenesis used iPSCs from patients with sporadic AD, familial AD,^67^ or Down's syndrome^68^ that differentiated neurons in 2D, but later studies showed that 3D models of familial AD were able to more accurately model Aβ and tau pathology in familial AD and Down's syndrome patient iPSCs, because of which patient‐specific models could be developed; these models are crucial to gain a full understanding of AD pathophysiology.^67^, ^68^, ^69^, ^70^, ^71^ These patient‐specific models clearly show pathological accumulation of amyloid β‐peptides and produce structures similar to amyloid plaques.^70^ Currently, attempts are being made to develop organoid models of the disease using multi‐tissue organoid models including astrocytes, oligodendrocytes, microglia, and neurons;^72^ organoid models developed using multiple tissues more closely resemble the pathological processes of AD, including tau protein hyperphosphorylation, neuroinflammatory activity, harmful Nitrogen Monoxide (NO) release to AD astrocytes and neurons, axonal segment fracture caused by neurotoxic activity, microglia recruitment, and β‐amyloid aggregation.^73^, ^74^ Researchers found that another nonneuronal factor in AD pathogenesis is the cerebral vasculature, and vascular single‐cell profiles from AD patients show that AD risk genes are abundantly expressed in human brain endothelial cells,^75^ so the inclusion of vascular cells in organoids and thus the study of some blood–brain barrier properties in the context of Aβ pathology is promising.^12^ Although these studies have reported visualization of promising primitive endothelial vasculature, it remains to be seen whether functional vascularization can be fully experimented with in vitro, and it can be argued that the absence of functional vascularization in organoids remains a major limitation of current research.^30^

### 4.5 Challenges and advancements in cerebral organoid research

At present, the main problems hindering the wide use of cerebral organoids in disease modeling include incomplete understanding of the cellular composition of cerebral organoids, the survival of neurons in cerebral organoids, and the reproducibility of cell types generated within a single organ‐like organ.^54^, ^76^ It is well known that organoid culture systems are now able to replicate the developmental stages of the fetal brain to a large extent, but the current inability of organoids to reach maturity in long‐term culture makes it difficult to model neurological disorders that affect the adult brain, such as Alzheimer's disease. Of course, many protocols have been optimized to overcome these obstacles, including pretreatment of organoids with brain‐derived neurotrophic factor to accelerate maturation, thus ensuring the survival of neurons,^54^ or the embedding of whole‐brain organoids in Matrigel containing iPSC‐derived endothelial cells to form vascularized organoids to transport oxygen and nutrients to support brain organoid maturation,^77^ but this is still far from enough. In addition, existing research shows that current neural models generally have batch syndrome, which leads to significant variability in the quality of the models,^78^, ^79^ thus affecting the reproducibility of experimental results in biological research. Also, somatic reprogramming of iPSC with the genome is currently unstable, particularly at known tumorigenic loci,^80^ while in aging‐related diseases that rely on proper modeling (e.g., AD and PD), it is difficult to model multiple genetic alterations in the transcriptional profile over time, making it difficult to simulate age‐related iPSC models.^81^ However, it is clear that the combination of biomaterials, nanotechnology, bioengineering, and other advanced technologies may enable organoids to break through the current bottleneck. Therefore, continued efforts should be focused on establishing the definition of standardized culture conditions and high‐quality production requirements for advancements in this field.

A few excellent breakthroughs have been made in the field of brain‐like organ research, such as fusion construction of cerebral organoids from different brain regions,^27^ formation of cerebral organoids containing mature neurons,^54^ vascularization of cerebral organoids,^12^ and so forth. These developments in the use of cerebral organoids are becoming increasingly important. Current developments in the field show the potential for mesodermal progenitors to develop into microglia‐like cells.^82^ Also, with the emergence of new models of cerebral organoids containing microglia,^83^, ^84^ we can more closely and better model the physiological microenvironment in vivo and thus better understand the pathological mechanisms of neurological diseases. Despite the limitations of the use of brain‐like organ technology, it is still hoped that it can provide more extensive and detailed dynamic images for studying the development of cerebral organoids in different diseases, so as to screen effective treatment and prevention strategies.

## 5 CONCLUSIONS

The field of cerebral organoid research has developed rapidly in recent years, with the United States taking the lead in contributions and increasing participation from Asian countries. Research is enhancing our understanding of the structure, function, genetics, and neurological disorders of cerebral organoid. Despite some technical limitations, 3D cerebral organoid models have significant research and application potential for the study of brain development as well as strategies for disease prevention and treatment.

## AUTHOR CONTRIBUTIONS

Bo‐Yan Luo participated in the design, data collection, visualization and review of this study. Ke‐Qian Liu wrote and revised the manuscript. Ji‐Sheng Fan contributed to the overall design, supervision of the work and formal analysis. All authors have read and approved the final version of the manuscript.

## CONFLICT OF INTEREST STATEMENT

The authors declare no conflict of interest.

## ETHICS STATEMENT

Not applicable.

## Data Availability

The data sets used and/or analyzed in the current study are available from the corresponding author on reasonable request.
